# Editorial: New strategies to inhibit cell death in myocardial ischemia-reperfusion injury: how to succeed? Volume II

**DOI:** 10.3389/fcvm.2023.1260800

**Published:** 2023-08-08

**Authors:** Sarawut Kumphune, Christophe Piot, Stéphanie Barrere-Lemaire

**Affiliations:** ^1^ Biomedical Engineering and Innovation Research Centre, Chiang Mai University, Chiang Mai, Thailand; ^2^ Biomedical Engineering Institute, Chiang Mai University, Chiang Mai, Thailand; ^3^ IGF, Université de Montpellier, CNRS, INSERM, Montpellier, France; ^4^ Département de Cardiologie Interventionnelle, Clinique du Millénaire, Montpellier, France

**Keywords:** ischemia-reperfusion injury, myocardium, cardioprotection, infarction, cell death, heart rate, shock waves, genetic manipulation

**Editorial on the Research Topic** New strategies to inhibit cell death in myocardial ischemia-reperfusion injury: how to succeed? volume II

## 1. Introduction

In 2023, myocardial infarction still ranks first for the worldwide cardiovascular mortality. Reperfusion therapy is the most effective treatment to reduce infarct size but, despite obvious benefits, it also has deleterious effects called ischemia-reperfusion (IR) injury for which no pharmacological treatment exists. In this topic are presented original approaches based on non-pharmacological cardioprotective strategies such as heart rate modulation, mechanical stimulation (shock waves) and finally genetic manipulations.

## 2. Natural protection

Cui et al. describes the cardioprotective effects of the main active component, peak 8 (P8) fraction, from Chick early amniotic fluid (ceAF). In a previous study, the authors discovered that ceAF is very effective in the rescue of myocardial injury, in mice and pigs models of IR injury (1). In the present topic, they demonstrate that P8 treatment was able to decrease apoptosis *in vitro* in human embryonic stem cell-derived cardiomyocytes subjected to hypoxia and reoxygenation. In addition, a 4 week-treatment by P8 allows to decrease both cardiomyocyte apoptosis and fibrosis deposit in an *in vivo* mouse model of myocardial infarction. Cardioprotection is mediated via an inhibition of NF-κB signaling and a downregulation of inflammatory cytokine expression. Altogether these results suggest that P8 from ceAF could be a promising therapeutic agent for ischemic cardiac injury.

Delgado-Betancourt et al. describe natural protection associated with decreased heart rates in mouse models subjected to both *ex vivo* and *in vivo* myocardial IR injury. The relationship between basal heart rate and infarct size was investigated using various genetically modified mouse strains. The results show that infarct size is closely correlated with heart rate recorded during the pre-ischemic and ischemic phases in all wild-type and mutant mice tested. These data demonstrate, for the first time, that heart rate *per se* is a major predictor of IR injury: the lower is heart rate, the greater being cardioprotection. Moreover, this study provides the evidences that inactivating Ca_v_1.3 channels can constitute a new potential therapeutic strategy to reduce IR injury and infarct size.

## 3. Mechanical protection

Mechanotransduction, a biological pathway to which many cells are sensitive to external mechanical-acoustic stimulation, can be activated by using shock wave (SW) therapy. Despite the regenerative effects of SW therapy on chronic myocardial ischemia, little is known about acute IR injury. In the present topic, Petrusca et al. investigated the cardioprotective effects of SW therapy in an open chest swine model via temporary coronary ligation followed by reperfusion. SW was applied to the ischemic myocardium for 2 min and pursued during 4 min after reperfusion. Cardiac magnetic resonance imaging was used to assess global function and quantify regional strain and myocardial oedema. The authors demonstrate a significant and early improvement of global systolic left ventricular function in treated animals. However, myocardial oedema was not reduced and only a trend to higher contractility in the border and remote segments was shown by regional imaging. This study provides, using mechanical stimulation, a new protective approach against IR injury.

## 4. Genetic manipulation and cardioprotection

Gene manipulation has been one of the prospective therapeutic approaches investigated for decades. Emerging evidence suggests that epigenetic regulation is intimately associated with the pathogenesis of myocardial IR injury, suggesting that epigenetics may serve as a novel therapeutic target to prevent IR injury (2). In the current topic, Boovarahan et al. demonstrated that global DNA hypermethylation in myocardial IR injury is associated with the downregulation of mitochondrial regulatory genes, antioxidant genes, and apoptotic regulatory genes, which mechanistically explain cellular apoptosis. Small molecules, which inhibit the epigenetic process of DNA methylation, may reduce IR injury. This article highlights how targeting epigenetic processes, especially by downregulation of genes involved in cellular injury, could provide cardioprotection against myocardial IR injury.

Mongkolpathumrat et al. reports here the *in vivo* overexpression of SLPI (secretory leukocyte protease inhibitor) encoding an endogenous protein with anti-protease activity in cardiac tissue. In a previous study, the cardioprotective activity of SLPI has been reported *in vitro*, *ex vivo*, and *in vivo* models (3, 4). In this study, the authors demonstrate that cardiac selective overexpression of the human SLPI (hSLPI) gene via adeno-associated viral gene delivery could reduce IR-induced cardiac injury and preserve ventricular function Therefore, gene therapy involving the overexpression of protease inhibitory proteins may be a novel modality and support an alternative therapeutic strategy for ischemic heart disease. However, long-term effects on cardiac remodelling and hypertrophy should be considered prior to proceed to real clinical utilization.

## Conclusion

The challenge of cardioprotection must be met given the still high morbidity and mortality rates for acute myocardial infarction suggesting that reopening the artery as recommended by the guidelines is not enough. The discovery of new targets opens up new opportunities.
